# National Health Insurance Role in Hospital Utilisation in Disadvantaged Areas: Evidence from Indonesia

**DOI:** 10.21315/mjms2024.31.6.16

**Published:** 2024-12-31

**Authors:** Ratna Dwi Wulandari, Leny Latifah, Agung Dwi Laksono, Wahyu Pudji Nugraheni, Tati Suryati, Tety Rachmawati, Diah Yunitawati, Rofingatul Mubasyiroh, Irfan Ardani, Asep Kusnali

**Affiliations:** 1 Faculty of Public Health, Universitas Airlangga, Surabaya, Indonesia; 2 National Research and Innovation Agency, Jakarta, Indonesia

**Keywords:** hospital utilisation, disadvantaged areas, National Health Insurance, healthcare evaluation, public health

## Abstract

**Background:**

The Indonesian government policy regarding obtaining universal coverage through National Health Insurance (NHI) is expected to increase public access to health service facilities, including in disadvantaged areas. This study analysed the role of NHI in hospital utilisation in underprivileged areas of Indonesia.

**Methods:**

Data from the 2023 National Socioeconomic Survey were used in this cross-sectional study that included 130,331 participants. Hospital utilisation was the dependent variable and NHI membership was the independent variable. Residence, age, sex, marital status, education, occupation, and wealth status were control factors. A multinomial logistic regression was employed in the final stage for data evaluation.

**Results:**

In 2023, the hospital utilisation rate in Indonesia’s disadvantaged regions was 1.5% and the percentage of NHI members was 74.5%. People with an NHI membership were 3.01 times more likely to utilise the hospital than those without [95% confidence interval (CI) 2.58–3.50]. Seven control variables related to hospital utilisation were identified, namely, residence type, age, sex, marital status, education level, employment status, and wealth status.

**Conclusion:**

This study concluded that NHI membership influenced hospital utilisation in disadvantaged areas of Indonesia. Individuals with NHI membership were three times more likely to visit hospitals.

## Introduction

The hospital referral system is an essential component of Indonesian healthcare. It is established in a hierarchy to ensure that each patient receives proper care at an appropriate level of service. Primary healthcare facilities are at the bottom of the system and provide initial contact and primary care. When a patient’s condition is too severe for these facilities to handle, they are sent to secondary or tertiary hospitals for specialised care. This system is designed to make the best use of resources and improve patient outcomes by sending patients to healthcare settings that best fit their needs (1). The effectiveness of a referral system depends on its ability to efficiently reduce disparities in healthcare access and manage patient burden. In Indonesia, several challenges remain such as unequal access to services and the accumulation of patients in specific hospitals, which can lead to overburden and decreased quality of services. Studies have shown that urban residents are more likely to use hospital services than rural residents, indicating this disparity in the referral system must be addressed (2).

Indonesia is the world’s largest archipelago and its different regions have different levels of health and economic well-being due to weather conditions and a lack of infrastructure. Disparities caused by growth also make it more difficult for people to access healthcare facilities (3). Regions with a good economic status tend to have better access to healthcare facilities. Hospital utilisation in Indonesia, including inpatient and outpatient services, has been the subject of several studies. In 2018, Indonesia’s national average inpatient hospital utilisation was 3.053%, and that for outpatients was 1.465% (4). This condition was better than in 2013, when the average utilisation was 1.6% for inpatients and 1.1% for outpatients (5). Hospital utilisation trends differ significantly between countries, typically reflecting each country’s healthcare structure and financial systems. Although the governments of Malaysia and Vietnam have enacted measures to attain equality, they encounter similar challenges in guaranteeing equal and fair access to health services in different areas of their countries (6, 7).

Previous studies have shown that various factors affect the extent to which healthcare is used. Using Andersen’s conceptual framework, researchers have categorised factors that influence healthcare use into three groups, i.e., need, allowing, and predisposing (8–11). According to research, age, education, knowledge, marital status, and sex are all associated with higher healthcare usage (9–11). Enabling factors are related to service use. Distance to a health facility, place of residence, insurance ownership, and income all contribute to the overuse and underuse of hospital services (8, 12, 13). Need parameters, such as disease severity and previous experience with healthcare utilisation, are also associated with the use of hospitals as referral healthcare institutions (8, 10, 11). Based on the above framework, geographical disparities and a national health insurance health protection programme are significant barriers and enabler to accessing hospital utilisation, respectively.

In Indonesia, the Ministry of Health Regulation No. 71 of 2013, along with its amendments, pertains to the utilisation of hospitals within the National Health Insurance (NHI) framework and constitutes a pivotal policy aimed at improving access to and quality of healthcare services for the population. This policy underscores the importance of fair access to healthcare services for all citizens. It underlines the imperative for collaboration among the central government, regional authorities, and private sector to guarantee effectiveness and efficiency in healthcare services (14, 15). Efforts to broaden healthcare service coverage are reflected in programmes, such as incentives for healthcare personnel and the development of healthcare facilities in these regions. In 2022, the National Social Security Council and the Health Social Security Agency (HSSA) registered 2,745 involved hospitals, including government and private hospitals, and main clinics [Advanced Referral Healthcare (ARHF)] in partnership with the HSSA (16). This number has increased since 2016 despite a decline in 2019. The ARHF-to-enrolled NHI participant ratio remained stable from 2016 to 2021, with a national ratio of 1 ARHF per 100,000 participants. However, the bed ratio in hospitals cooperating with the HSSA is 2 per 1,000 participants, which falls short of the World Health Organization recommendation of 5 per 1,000 people. A significant aspect of this policy is the regulation of healthcare financing, including hospital costs, through the NHI mechanism. In practice, the NHI allows participants, including those residing in underserved areas, to receive services at hospitals participating in the programme (17). The NHI encourages hospitals to expand their reach and improve the quality of healthcare services, including in underserved areas, through incentives and regulations, some of which are accommodated in the Ministry of Health Regulation No. 21 of 2020 from the Ministry of Health’s Strategic Plan.

Based on this background information, this study analysed the role of the NHI in hospital utilisation in Indonesia’s disadvantaged areas.

## Materials and Methods

### Data Source

This study used secondary data obtained from the 2023 National Socioeconomic Survey, which was a nationwide cross-sectional survey conducted by Indonesian Statistics. The poll gathered data in March 2023.

The 2023 National Socioeconomic Survey comprised the entire population of Indonesian households. This study covered a sample size of 345,000 households distributed among 34 provinces and 514 districts/cities in Indonesia. The survey used a systematic sampling method to select a sample of 10 homes in each census block, which were selected using probability proportional to size. The total number of census block samples used for the survey was 34,500. The survey conducted stratification at the census block and household levels within specific census blocks to obtain a sample that accurately represented the population. Census block stratification involved explicitly grouping the complete population of regular census blocks from the 2020 Population Census according to urban or rural classifications. Implicit stratification was based on the education level for the head of household (18).

This survey included all individuals aged ≥15 years who resided in Indonesia’s underprivileged areas. The sampling methods determined the sample size, which consisted of 130,331 participants.

### Setting

The study examines the utilisation of hospitals in Indonesia’s disadvantaged regions. Presidential Regulation Number 63 of 2020, titled “Determining Underdeveloped Regions for 2020–2024”, defines the limits of economically underprivileged regions. According to the regulation, underdeveloped areas in Indonesia include 62 provinces in 11 provinces: North Sumatera Province (Nias, South Nias, North Nias, West Nias), West Sumatera Province (Mentawai Islands), South Sumatera Province (North Musi Rawas), Lampung Province (West Pesisir), West Nusa Tenggara Province (North Lombok), East Nusa Tenggara Province (West Sumba, East Sumba, Kupang, East Timor Tengah, Belu, Alor, Lembata, Rote Ndao, Central Sumba, Southwest Sumba, East Manggarai, Sabu Raijua, Malaka), Central Sulawesi Province (Donggala, Tojo Una-una, Sigi), Maluku Province (West Maluku Tenggara, Aru Islands, West Seram, East Seram, Southwest Maluku, South Buru), North Maluku Province (Sula Islands, Taliabu Island), West Papua Province (Wondama Gulf, Bintuni Gulf, South Sorong, Sorong, Tambrauw, Maybrat, South Manokwari, Arfak Mountains), and Papua Province (Jayawijaya, Nabire, Paniai, Puncak Jaya, Boven Digoel, Mappi, Asmat, Yahukimo, Bintang Mountains, Tolikara, Keerom, Waropen, Supiori, Great Mamberamo, Nduga, Lanny Jaya, Central Mamberamo, Yalimo, Puncak, Dogiyai, Intan Jaya, Deiyai).

### Outcome Variable

Hospital utilisation was the dependent variable, which pertained to the degree individuals used hospitals for outpatient and inpatient treatments. Hospital utilisation was categorised as unused or used. In contrast, the study focused on outpatient hospitalisations that occurred the month before while it examined inpatient hospitalisations that occurred in the previous year. The study requested that individuals recollect outpatient and inpatient incidents (19).

### Exposure Variable

NHI membership was an independent variable in the analysis. NHI comprises all forms of membership, including mandatory enrolment for public servants, police, and army personnel, and coverage provided by companies or through government support. NHI membership was classified as either “No” or “Yes”.

### Control Variables

Seven control factors were incorporated, namely, residence type, age, sex, marital status, education level, employment status, and wealth status. The survey classified residential areas into two distinct categories, i.e., urban and rural. Furthermore, the survey utilised the urban-rural classification criteria provided by Indonesian Statistics.

The participants’ ages were determined by their most recent birthday. Sex was classified into males and females. Marital status had three categories, i.e., never married, married, and divorced or widowed.

The term “respondent’s education” referred to current academic credentials grouped into four levels of education, namely, primary school, middle school, high school, and university. Employment status was either unemployed and employed.

The survey utilised the wealth index formula to determine the participants’ status. The survey calculated the wealth index using a weighted average of household expenses. The poll determined the wealth index by considering primary household expenditures such as health insurance, food, housing, and other miscellaneous items. Furthermore, the poll classified the income index into five categories, i.e., least affluent, less affluent, middle, more affluent, and most prosperous (20).

### Data Analysis

At the beginning of the sample period, the chi-squared test was used to compare the two categories of variables. Concurrently, *t*-tests were used to assess continuous age variables. A collinearity test was used to confirm that the independent variables in the final regression model were not strongly associated. The final step in the study involved the use of a multinomial logistic regression analysis. This study utilised a previously established method to analyse the multivariate correlations between all independent variables and hospital utilisation. Statistical Package for the Social Sciences (SPSS) version 26 (IBM Corp., Armonk, NY, US) was used for the statistical analyses.

## Results

**Figure 1:**
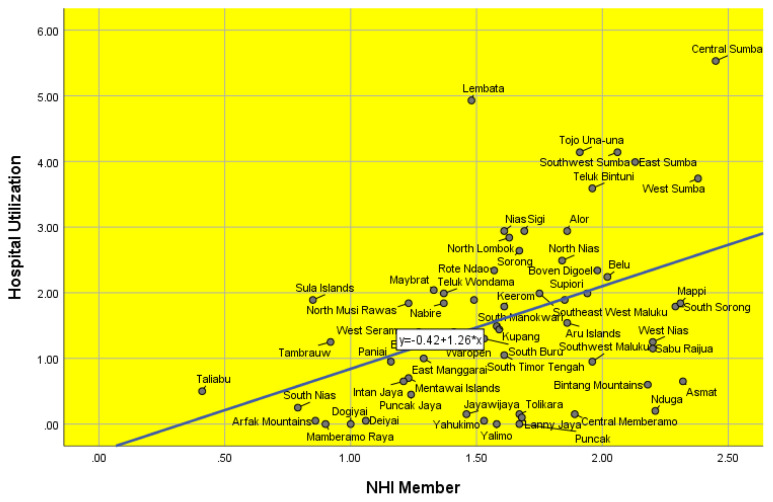
Scatter plot of the proportion of NHI members and hospital utilisation in disadvantaged areas by the regency in Indonesia in 2023 Source: Visualisation by the authors

This study found that the hospital utilisation rate in Indonesia’s disadvantaged regions in 2023 was 1.5%. The percentage of NHI members was 74.5%. Table 1 lists the characteristics of the respondents. Figure 1 displays the scatter plot for the proportion of NHI members and hospital utilisation in the disadvantaged areas by regency in Indonesia in 2023. A positive relationship between these two factors was indicated (Figure 1). The higher the proportion of NHI members, the higher the hospital utilisation in the regency.

Table 2 presents the results of bivariate analysis. NHI members used hospitals more than uninsured individuals. Rural areas were the dominant residence type in both NHI member groups. People with a NHI membership had a mean age older than that of those without a NHI membership. Males had a higher ratio than females in both NHI membership groups.

The data in Table 2 show that regarding marital status, never married dominated in both groups of NHI membership. Based on education level, primary school dominated in both groups of NHI membership. Meanwhile, unemployed dominated for those with all types of NHI membership. The poorest category of wealth status dominated in the two groups of NHI membership.

Subsequently, a collinearity test was performed. The test results suggested no noticeable connection between the independent variables. The tolerance value was more significant than 0.10, whereas the variance inflation factor value for each variable was less than 10.00. The analysis revealed no evidence of multicollinearity in the regression model, suggesting that the test’s basis for generating conclusions was strong.

Table 3 presents the findings of the multinomial logistic regression analysis on hospital use in Indonesia in 2023. Based on NHI membership, individuals with an NHI membership were 3.01 times more likely to utilise the hospital than those without (95% confidence interval [CI] 2.58–3.50).

This study demonstrated a correlation between hospital utilisation and six control factors. Based on residence type, individuals living in rural areas were 0.58 times less likely to use the hospital than those in urban areas (95% CI 0.51–0.65). Five demographic factors were linked to hospital utilisation in disadvantaged communities in Indonesia, namely, age, sex, marital status, education level, and employment position. Furthermore, based on wealth status, Table 3 shows that the wealthier people were the more likely they are to use the hospitals.

## Discussion

This study found that individuals who joined the NHI programme changed how often they used hospitals in Indonesia’s poorer areas. There are several reasons people were more likely to visit the hospital since the NHI programme started, especially in regions that are not well developed. The NHI can help people who have trouble paying for hospital care. People with a poorer status who sign up for NHI can receive medical care without spending much on hospital stays, treatments, and procedures (17, 21–24). The increase in hospital utilisation may also be attributed to the integration of health promotion initiatives within the NHI programme, which aims to enhance knowledge of healthcare needs (25). Hospital services need improvement and capacity must to be increased to ensure that people receive good care in places that are not well developed and do not have adequate healthcare infrastructures. These changes may include training healthcare workers, improving medical facilities and equipment, and establishing quality assurance protocols to ensure that patients receive quick and reasonable care (26, 27)

The results of this study revealed that individuals residing in disadvantaged areas of Indonesia were three times more likely to have NHI membership than those who did not. The higher likelihood of hospital utilisation among individuals enrolled in the NHI in underserved areas proves the vital role of the NHI (23, 28). The NHI policy ensures equitable access to healthcare for residents in underserved areas (17, 29), including hospitals. Earlier studies attributed the increase in hospital utilisation to the implementation of health insurance ownership (4, 21, 30). Health insurance schemes in low- and middle-income countries generally increase access to healthcare facilities (30). In Malaysia, health insurance ownership determines access to healthcare, although it does not influence frequency of use (7). A study of four states in the US also found no increase in hospital utilisation after the expansion of Medicaid; however, uncompensated costs decreased significantly (31). Thus, the specific impact of the NHI might differ based on various circumstances such as the healthcare policies and systems of different nations.

The utilisation of hospital services is a benchmark for the success of a healthcare referral system. Several challenges remain, such as unequal access to services and the accumulation of patients in certain hospitals (2). In Indonesia, the NHI policy aims to ensure equitable access to healthcare for residents in underserved areas (17, 29), including hospitals. The equity aim aligns with Health Law No. 17 of 2023 and Government Regulation No. 78 of 2014 to accelerate the development of underserved areas, emphasising the importance of considering primary healthcare needs in underserved areas in healthcare facility development, including but not limited to healthcare financing patterns (32). A significant aspect of this policy is the regulation of healthcare financing, including hospital costs, through the NHI mechanism. In practice, the NHI allows participants, including those residing in underserved areas, to receive services at hospitals participating in the programme (17). The NHI encourages hospitals to expand their reach and improve the quality of healthcare services, including in underserved areas, through incentives and regulations, some of which are accommodated in the Ministry of Health Regulation No. 21 of 2020 on the Ministry of Health’s Strategic Plan.

In this study, individuals living in rural areas were less likely to use hospital services than those living in urban areas. Rural areas often require better access because of geography and gaps in the distribution of resources (4). Vietnam have shown that significant strides in healthcare access have been achieved, although urban-rural disparities still exist (6). There are few health workers and healthcare facilities in rural areas, particularly for referral healthcare (4). Patients in rural areas rely mainly on essential healthcare services and cannot receive as much specialised medical care as those in cities where hospitals are better equipped (6). Higher hospital utilisation rates have been observed in cities with better healthcare facilities (33). How well a referral system works depends on how well it can level the playing field regarding healthcare access and handling patient burden. When new rules and funds for healthcare are made, this gap should be prioritised (2).

This study revealed five demographic factors linked to hospital utilisation i.e., age, sex, marital status, education level, and employment position. Healthcare utilisation increases with age (34). Adults are highly susceptible to various chronic illnesses. As they age, they tend to develop multiple diseases (35). In this study, females were less likely to visit hospitals than men. Similar results show that males have a greater hospital utilisation rate because of the influence of economic status and health conditions (36). A South African study reported contrasting results (34). This findings regarding marital status in this study are consistent with a survey conducted in Maluku Province that found hospital utilisation is more common among married or divorced individuals than among single individuals (37, 38). Similar to this study, research in South Africa demonstrated that the higher the education level, the higher the utilisation of health services (34). People with more education use health services more frequently because they have a better awareness of health-related aspects (39). Educated people devote more attention to health problems and often take preventative actions to improve health outcomes, which reduces utilisation of health services (40). Hospital utilisation was lower among employed individuals than among unemployed individuals. Employed people may be less likely to go to the hospital unless they have to because of work obligations and the risk of losing income or job security that comes with taking time off. Unemployed people may be more stressed, live in poor conditions, or have trouble obtaining healthy foods. These factors can hurt health and cause more people to visit hospitals. People with jobs may do healthier things such as eating well and exercising regularly, which can lower their need for hospital care (41).

This study showed that the wealthier the participants were, the more likely they were to use hospitals. Wealthier individuals can purchase health insurance or pay out-of-pocket for their required treatments and can access medical treatment; others can have financial barriers that prevent access to health services. Wealthy people with higher education levels are more proactive about their health, seeking preventative treatment and undergo regular examinations, which can lead to early disease identification. Affluent people are more likely to utilise hospitals that offer high-quality services, including access to advanced medical equipment (42, 43). Furthermore, research in Ghana found that the advantages derived from commercial and public healthcare services disproportionately favour wealthier individuals over less affluent individuals (44).

### Strength and Limitation

The study analysed extensive data to present facts on a nationwide scale. However, the study analysed secondary data, which implies that it only considers relevant aspects for examination. Prior research has not investigated supplementary variables associated with hospital utilisation, such as the length and cost of transportation to the hospital and the particular nature of the illness (2, 45–47).

## Conclusion

The study concluded that NHI membership influenced hospital utilisation in disadvantaged areas in Indonesia. Individuals with NHI membership were three times more likely to visit hospitals. Therefore, ensuring universal health coverage for those residing in the underprivileged regions of Indonesia is essential for achieving health equity, particularly for accessing the referral healthcare system. The threefold increase in hospital visits among NHI members indicated that having insurance made health care access more equal. This situation is something that policymakers should prioritise when developing health programmes that aim to reduce differences regarding who can receive health care.

## Figures and Tables

**Table 1:** Characteristics of respondents (n = 130,331)

Characteristics of respondents	n	%
Hospital utilisation		
Unutilised	128,324	98.5
Utilised	2,007	1.5

NHI member		
No	33,198	25.5
Yes	97,133	74.5

Residence type		
Urban	12,821	9.8
Rural	117,510	90.2

Age	130,331	100.0

Gender		
Male	66,307	50.9
Female	64,024	49.1

Marital status		
Never Married	67,642	51.9
Married	55,655	42.7
Divorced/widowed	7,034	5.4

Education level		
Primary school	87,406	67.1
Junior high school	18,983	14.6
Senior high school	17,431	13.4
College	6,511	5.0

Employment status		
Unemployed	74,125	56.9
Employed	56,206	43.1

Wealth status		
Poorest	53,131	40.8
Poorer	29,032	22.3
Middle	19,538	15.0
Richer	15,938	12.2
Richest	12,672	9.7

**Table 2:** Bivariate analysis (n = 130,331)

Demography characteristics	NHI member		*p*-value

	No (n = 33,012)	Yes (n = 97,133)	
Hospital utilisation			< 0.001
Unutilised	99.4	98.1	
Utilised	0.6	1.9	

Residence type			< 0.001
Urban	9.3	10.0	
Rural	90.7	90.0	

Age (mean)	(23.57)	(29.94)	< 0.001

Gender			
Male	51.0	50.8	
Female	49.0	49.2	

Marital status			< 0.001
Never in union	61.4	48.7	
Married	34.0	45.7	
Divorced/widowed	4.6	5.7	

Education level			< 0.001
Primary school	73.9	64.7	
Junior high school	12.5	15.3	
Senior high school	10.7	14.3	
College	2.9	5.7	

Employment status			< 0.001
Unemployed	66.7	53.5	
Employed	33.3	46.5	

Wealth status			< 0.001
Poorest	48.6	38.1	
Poorer	23.1	22.0	
Middle	13.7	15.5	
Richer	9.0	13.3	
Richest	5.6	11.1	

**Table 3:** The findings of the multinomial logistic regression study on hospital use in Indonesia in 2023 (n = 130,331)

Predictor	Utilised hospital			

	Adjusted odds ratio	95% CI		*p*-value

		Lower bound	Upper bound	
NHI membership: No (ref.)	–	–	–	–
NHI membership: Yes	3.01	2.58	3.50	<0.001
Residence: Urban (ref.)	–	–	–	–
Residence: Rural	0.58	0.51	0.65	<0.001
Age	0.005	0.004	0.007	<0.001
Gender: Male (ref.)	–	–	–	–
Gender: Female	0.90	0.81	0.99	0.038
Marital: Never in union (ref.)	–	–	–	–
Marital: Married/living with partner	3.43	3.06	3.86	<0.001
Marital: Divorced/widowed	3.79	3.15	4.56	<0.001
Education: Primary school (ref.)	–	–	–	–
Education: Junior high school	0.96	0.84	1.10	0.539
Education: Senior high school	1.25	1.11	1.42	<0.001
Education: College	1.74	1.48	2.04	<0.001
Employment: Unemployed (ref.)	–	–	–	–
Employment: Employed	0.39	0.34	0.43	<0.001
Wealth: Poorest (ref.)	–	–	–	–
Wealth: Poorer	1.28	1.12	1.45	<0.001
Wealth: Middle	1.45	1.27 1.67		<0.001
Wealth: Richer	1.56	1.36	1.79	<0.001
Wealth: Richest	1.71	1.47	1.98	<0.001

## Data Availability

The data supporting this study’s findings are available from the Indonesian Statistics. Nonetheless, restrictions apply to the availability of these data, which were used under licence for the current research and are not publicly available. The data are available to researchers who need it. They can submit a request to Indonesian Statistics via https://silastik.bps.go.id/v3/index.php/site/login/.
